# Biomarker Application for Precision Medicine in Stroke

**DOI:** 10.1007/s12975-019-00762-3

**Published:** 2019-12-18

**Authors:** Alexis N. Simpkins, Miroslaw Janowski, Helieh S. Oz, Jill Roberts, Gregory Bix, Sylvain Doré, Ann M. Stowe

**Affiliations:** 1 Department of Anesthesiology, University of Florida, Gainesville, FL USA; 2 Department of Diagnostic Radiology and Nuclear Medicine, University of Maryland, Baltimore, Baltimore, MD USA; 3 Department of Internal Medicine, University of Kentucky, Lexington, KY USA; 4 Department of Neurosurgery, University of Kentucky, Lexington, KY USA; 5 Center for Advanced Translational Stroke Science, Lexington, KY USA; 6 Department of Neurology, University of Kentucky, Lexington, KY USA; 7 Clinical Neuroscience Research Center, Tulane University, New Orleans, LA USA; 8 Department of Neurosurgery, Neurology, Tulane University, New Orleans, LA USA; 9 Department of Neurology, Psychiatry, Pharmaceutics, Neuroscience, University of Florida, Gainesville, FL USA

**Keywords:** biomarker, ischemic stroke, precision medicine, clinical trials, hemorrhagic stroke, 'omics', big data

## Abstract

Stroke remains one of the leading causes of long-term disability and mortality despite recent advances in acute thrombolytic therapies. In fact, the global lifetime risk of stroke in adults over the age of 25 is approximately 25%, with 24.9 million cases of ischemic stroke and 18.7 million cases of hemorrhagic stroke reported in 2015. One of the main challenges in developing effective new acute therapeutics and enhanced long-term interventions for stroke recovery is the heterogeneity of stroke, including etiology, comorbidities, and lifestyle factors that uniquely affect each individual stroke survivor. In this comprehensive review, we propose that future biomarker studies can be designed to support precision medicine therapeutic interventions after stroke. The current challenges in defining ideal biomarkers for stroke are highlighted, including consideration of disease course, age, lifestyle factors, and subtypes of stroke. This overview of current clinical trials includes biomarker collection, and concludes with an example of biomarker design for aneurysmal subarachnoid hemorrhage. With the advent of “-omics” studies, neuroimaging, big data, and precision medicine, well-designed stroke biomarker trials will greatly advance the treatment of a disease that affects millions globally every year.

## Introduction

Despite the advances in the care of patients, stroke remains one of the leading causes of death and long-term disability in adults [1–3]. The approximate lifetime risk of stroke in adults older than 25 worldwide is approximately 25% [4], with 24.9 million cases of ischemic stroke and 18.7 million cases of hemorrhagic stroke reported globally in 2015 [1]. Although the age-adjusted incidence of stroke may be declining, the prevalence of the disease and the global health burden will rise as the size of the aging community increases. In addition, the prevalence of stroke is also increasing in younger populations [4]. This will affect patients who would otherwise be a part of the workforce, with a potential loss of income from long-standing disability that also impacts the family unit and the community as a whole [3]. While middle and low socioeconomic regions are particularly impacted by stroke [2, 4], it has a significant influence on all communities, even when care is given in the most advanced and comprehensive clinical setting. Despite the most advanced acute interventions, a significant number of patients will suffer residual disability as a consequence of stroke. The HERMES meta-analysis of the highly successful thrombectomy trials found that 54% of patients who received thrombectomy still had either persistent long-term disability or died [5, 6]. Stroke-related healthcare costs are expected to climb approximately $3 billion per year, with an estimated total healthcare cost of $94.3 billion per year expected by 2035 in the USA [1]. Thus, stroke remains a formidable disease that will continue to impact society in the absence of effective prevention, new acute therapeutic modalities, and enhanced interventions for long-term recovery.

## Current Challenges in Defining Ideal Biomarkers for Stroke

### 1. Biomarker utilization

The development and honing of individualized care for patients require the ability to account for the multitude of variables that influence patient outcome. Stroke is a heterogeneous group of disorders subdivided by many variables such as ischemic versus hemorrhagic, stroke etiology, size, location, and severity. As a result, it is crucial to have methods that allow extensive clinical phenotyping. One way to accomplish this is through biomarkers. A biomarker can be created from clinical data points, fluid, or tissue analysis (for various “-omics,” e.g., transcriptomics, lipidomics, proteomics, or metabolomics), clinical outcome scales, toxicology, imaging, physiologic testing, histology, microbiome analysis, and biochemical studies, to name a few. These biomarkers can improve diagnostic precision, predict clinical outcomes, select patients for clinical trials, monitor disease progression, and identify new therapeutic targets [7, 8]. Biomarkers could also precisely determine beneficial, futile, or harmful treatments [9]. For example, biomarkers could accurately predict the risk of non-aneurysmal rupture and subarachnoid hemorrhage (SAH) [10].

### 2. Ideal characteristics of an ideal biomarker

Ideal biomarkers in stroke should differentiate between patients with and without the disease state or clinical outcome of interest with high sensitivity and specificity. For stroke, ideal biomarkers should be cost-effective, quickly obtainable without interfering with administration of acute therapies, and non- or minimally invasive [7, 8, 11]. In order for biomarkers to be universally applicable, methods for data acquisition should be standardized and realistically plausible even in settings with limited resources [12]. Biomarker utilization should allow for avoidance of duplication of efforts among researchers. Obtaining and sharing the biomarker data should be possible while maintaining human subjects’ protection. In addition, an ideal biomarker would have a signal that is not heavily influenced by confounders like gender, age, ethnic background, diet, medications, circadian rhythm, environmental exposures, and other medical comorbidities.

### 3. Challenges in biomarker utilization

Stroke is very complex, so it is not likely that one biomarker will be the gateway to precision medicine for treatment. This requires a paradigm shift away from the hunt for the single biomarker that can direct clinical care and research for all. The challenge is in the selection of biomarkers with high sensitivity and specificity among the many variables that may affect outcomes. Sensitivity and specificity are a challenge with stroke biomarkers, in particular, because the stroke itself triggers multi end-organ stress that results in dynamic changes in expression and cytokine release among the background biological signals that will be unique to each person. Direct central nervous system sampling is often not possible unless a patient requires a surgical procedure that would provide that access for clinical reasons such as open aneurysm clipping, intraventricular drain placement, or specialized intracranial pressure monitoring. Thus, biomarker analysis is limited to mostly indirect measures of brain activity, imaging, blood, other body fluid analysis, and clinical data for prediction models. Variables such as sex, age, ethnic background, diet, medications, environmental exposures, and other comorbidities may confound biomarkers. Confounders affect the signal-to-noise ratio and thus the sensitivity, specificity, and predictive value of the biomarker [7, 8]. It is more likely that multiple biomarkers or panels from “Big Data” could improve precision [8, 12–16]. Big Data in stroke effectively aids in the identification of relevant biomarkers from the myriad possibilities [17]. In fact, Big Data recently identified the utter lack of African patient data in 31 stroke-related genome-wide association studies that identified genetic determinants of several stroke subtypes [18], which will be an important requirement for any effective population-based biomarker. Although useful, registries and clinical trial data may exclude the identification of confounding variables [12]. Partnering with large data analytic companies for Big Data analysis can provide insight on key variables that may otherwise not be captured. Challenges faced in rehabilitation research also demonstrate variability in the types and assessment of biomarkers for tracking clinical outcome, objective assessment of recovery limits, and replication of research findings [19]. In addition, efforts are needed to harmonize biomarker assessment and improve secured portability of data for analysis between research collaborators [12]. Last, the importance of finding cytoprotective agents in the post-endovascular therapy era will require effective biomarkers [8, 20]. Neuroprotective agents thus far have failed to effectively translate to clinical application, but appropriately selected biomarkers may revive this effort [13]. Because of these challenges, any proposed biomarker would need to be cross-validated [21].

Thus, an ideal biomarker, or most likely group of biomarkers, will successfully achieve the following goals:

1. Stratify patients who will respond better to treatments, especially with regard to relevant clinical trials;
2. Identify which treatment(s) to apply to a specific patient;
3. Monitor the response to a treatment for information on whether a treatment should be changed to improve efficacy;
4. Advance the therapeutic field toward using individualized patient outcomes as endpoints.

In this review, we summarize biomarkers throughout the continuum of stroke care, the implications of biomarker use in clinical trials, the utility of clinical biomarkers in reverse translation to preclinical studies, and the coordination and planning necessary for successful biomarker development.

## Biomarkers in the Context of Disease Course

Very simple blood biomarkers are often used in stroke care (e.g., blood glucose, cholesterol, and hemoglobin A1c [22]) in addition to clinical and physiologic data and imaging, but the requirement for more advanced panels of biomarkers has been recently recommended [23]. Although current biomarkers tune therapy at the population level, the true value and opportunity for a paradigm shift uses technological advances made feasible through implementation of -omics technology. This would allow medical personnel to predict individual patient outcomes based on personalized biomarkers, which would be particularly effective in long-term assessments as each patient is his/her/their own control. This would also shift clinical research from large, expensive randomized clinical trials to smaller, more tailored groups of patients using predicted outcomes as the benchmarks.

From the perspective of biomarker research in stroke, the management of disease can be divided into three phases: pre-stroke (potentially years prior), stroke (both pre-hospital and in-hospital phases), and post-stroke phase of recovery and rehabilitation. The differences between these phases stem from population access, cost, speed, and depth of analysis. Stroke biomarker research can and should be performed in each phase independently and pursued separately. Phase-specific biomarker research includes:

1. Pre-stroke phase

The research on comprehensive stroke biomarkers in the pre-stroke phase remains in its infancy. This phase must be a cost-effective and population-based endeavor that includes -omics, activity, environment, and electronic health record. Optimally, data collection should be continuous over a long period to optimize within-patient controls and comparisons with patients who do not develop the disease. Primary questions include whether the patient will experience stroke, prediction of downstream sequela related to the stroke, and treatment responses. Such answers can be delivered only through recruitment of entire populations (e.g., Framingham [24] or Baseline [25] studies), though studies in stroke-prone populations (e.g., hypertensive, obese) based on global statistical analysis are also needed [26]. More recently, reports of increased risk of adverse events with aspirin when used for cardiovascular disease prevention in patients without symptomatic cardiovascular disease [27] highlight the need for patient-individualized interventions. Better identification of patients who could suffer from stroke within a whole population would have a tremendous impact on healthcare, as the new algorithm would better identify high-risk stroke groups on the level of individual patients. This could introduce the concept of a “biomarker passport,” which would provide patient-specific guidelines and expected outcomes readily available to the care management team prior to the first or recurrent stroke for optimization of stroke prevention. An example is the Disparities in Transition of Care After Acute Stroke Hospitalization, in which enrolled patients are followed and multidomain Big Data analysis, including clinical, environmental, and social determinants data, will be used to identify key variables associated with post-discharge outcomes in stroke patients [28]. This also would have potential impact on management in the acute setting. For instance, knowledge about collateral circulation is essential in making decisions regarding mechanical thrombectomy. It would be highly valuable to have that information before a stroke occurs as a “biomarker passport.” Having biomarker passport a priori would open the window of opportunity for novel therapeutic options prior to the onset of ischemia specifically to optimize collateralization.

Currently, we predict the risk of the whole population based on specific classifiers, but these data are still not compelling for individuals. Therefore, pre-stroke biomarker research through application of biomarkers and Big Data science would identify patient-specific risks to various pathological conditions, predicted severity, and pace of progression. Because of the involvement of large populations in pre-stroke phase biomarker development, these biomarkers should be relatively low cost and multimodal. However, there is no need for speed of data collection and processing. Therefore, the data can be processed by large medical centers and include the genomic background as well as lifestyle activity and environment (e.g., light intensity, pollution). In addition, easily available biological materials such as saliva or urine are preferable; however, in the early phase of research, these samples should be checked against blood and cerebral spinal fluid (CSF) analysis, which would be more difficult to obtain for high-frequency longitudinal analysis. The breadth of obtainable information could counterbalance this, while adding other -omics could be beneficial if cost was not prohibitive. Finally, having individualized information about stroke risk can improve stroke systems of care. The “biomarker passport” can be used to assist patients with making tailored lifestyle changes for stroke prevention and when the data are combined with other patients, it can also be used to ensure that resources are appropriately provided to facilitate these endeavors. For example, having these data can be useful for determining the impact of having easily accessible healthy food options, clinics, pharmacies, and parks and recreations, to name a few. In addition, it can be used to coordinate transportation and triage models for emergency medical services and hospitals.

2. Stroke phase

**Fig. 1:**
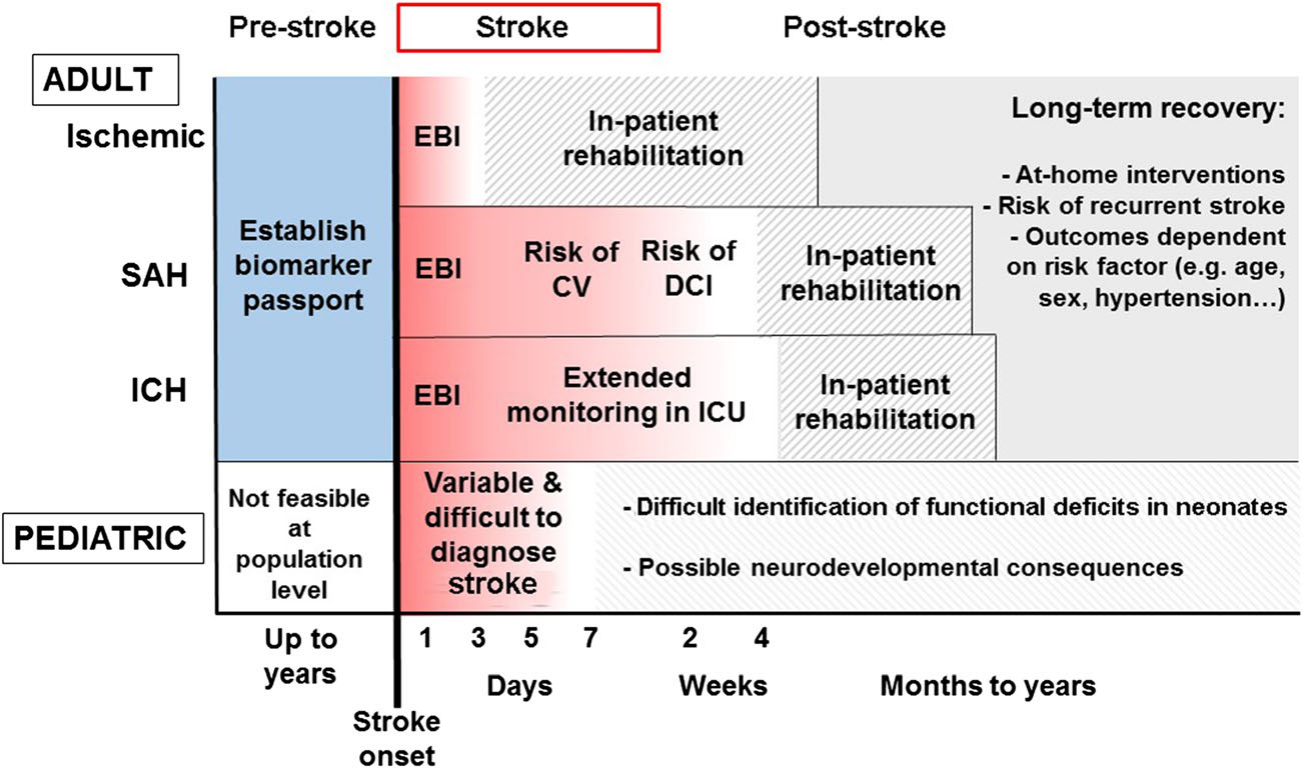
Phases of stroke-related biomarker study. The diagram outlines how biomarker studies should be tailored to both stroke subset (y-axis) and phase of stroke (x-axis). The phases include pre-stroke (blue), stroke (red) dependent on subtype, and post-stroke phase (gray). Current challenges for pediatric stroke biomarker trials are listed in the bottom row. SAH, subarachnoid hemorrhage; ICH, intracerebral hemorrhage; EBI, early brain injury; CV, cerebral vasospasm; DCI, delayed cerebral ischemia; ICU, intensive care unit

**Fig. 2:**
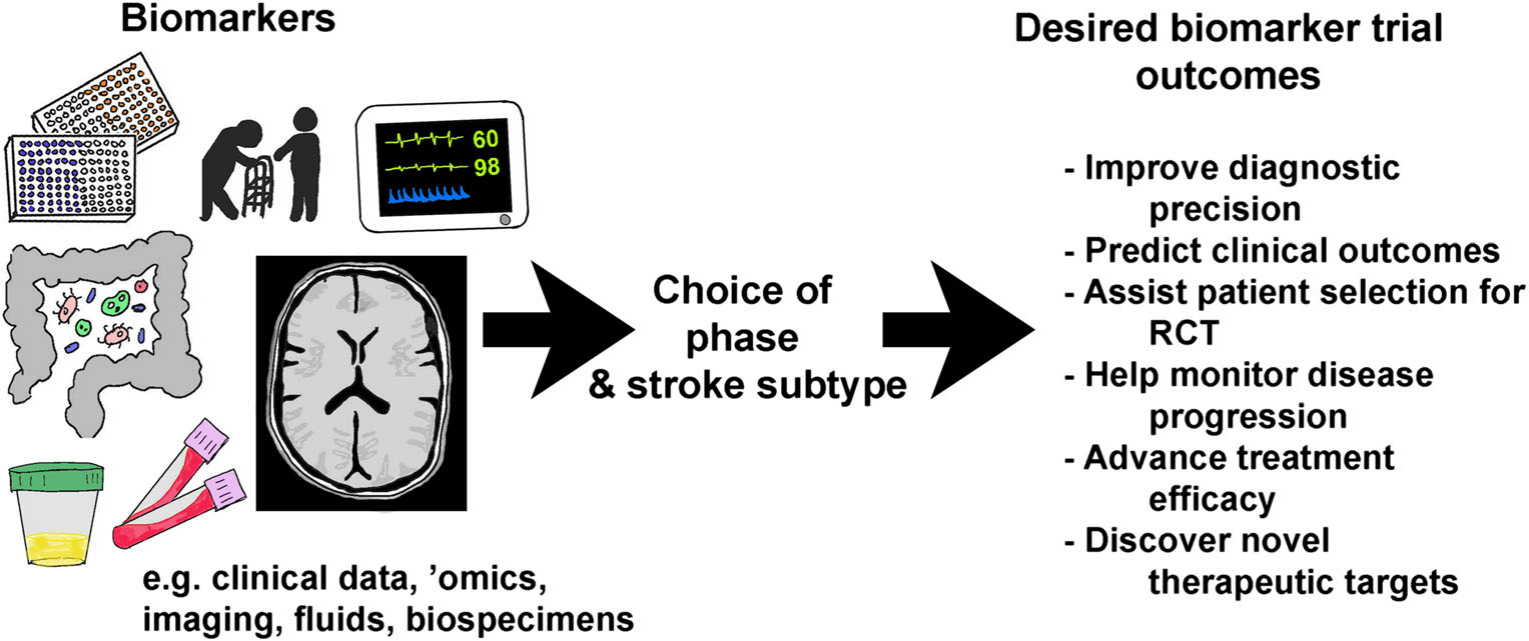
Schematic for optimizing stroke biomarker trials. Biomarkers can be identified in each phase of stroke (pre-stroke, stroke, post-stroke) and stroke subtype (ischemic, subarachnoid hemorrhage, intracerebral hemorrhage) to support precision medicine for the individual patient. RCT, randomized controlled trial

Biomarker development in this phase should include what is clinically considered the hyperacute phase (< 6 h after stroke onset). This phase would be both pre-hospital when a person arrives at the emergency department, as well as the in-hospital acute phase (6–72 h after stroke onset), although note that these time frames relate to ischemic stroke. Within the 6–24 h, the latter phase is of particular importance for ischemic stroke, as this is within the extended time window, which some patients may be eligible for endovascular therapy [29, 30]. There is also more compelling evidence suggesting that selected patients may benefit from receiving thrombolysis [31–33]. During the first several days after symptom onset for ischemic and hemorrhagic stroke, care is focused on medical stabilization. After medical stabilization, preparation of the patient for rehabilitation is a priority until new interventions are developed. This phase of biomarker development is dependent on stroke etiology, size, and medical complications (Fig. 1). For example, large ischemic strokes, SAH, and intracerebral hemorrhage (ICH) patients may have complications at later times (> 72 h after stroke onset), extending the window for intensive therapy. Also, the pathophysiology of hemorrhagic stroke differs from ischemic; thus, we should consider biomarker development for SAH/ICH separately from ischemic stroke, as outlined below. Finally, biomarkers may change dramatically in a short window of time during the hyperacute and acute phases compared with other phases, which is a challenge for identifying optimal treatment windows (Fig. 2).

The occurrence of stroke typically prompts evaluation for immediate therapeutic intervention. The biomarker passport could play an invaluable role in streamlining stroke care; however, acute phase biomarkers are a desirable addition. In this phase, only patients with stroke can be studied, which limits study recruitment for ideal controls. Moreover, likely the most valuable data from advanced imaging and monitoring would come from comprehensive stroke centers in which cutting-edge management is available. In this intensive period, timing is also critical. Therefore, output from biomarkers should be very fast or even instant. Thus, biomarker development should focus on speed and accuracy, and less on cost. In fact, there are already biomarkers in acute stroke care (e.g., imaging) that determine indications for thrombectomy and mismatch between clinical deficit and infarct size [29]. However, there are still opportunities for improvement, better to guide the therapeutic processes in this intensive phase, and identify mechanisms that may be adjunctive therapeutic targets to thrombectomy.

3. Post-stroke phase

After medical stabilization, the requirements for biomarkers change, as does the utility of particular biomarkers. Unfortunately, the patient population is now further limited to survivors, and the application of biomarkers in this phase is more challenging due to difficulties in finding effective interventions to enhance neuroplasticity and recovery. There is already a known rule of 70% recovery on the population-based level, which could be used as a benchmark for population studies [34]. However, at the single-patient level, there is high variability preventing the use of patients as their own controls [35]. Therefore, identifying better “predictors” of recovery on a patient-based level would be the most beneficial, particularly through next-generation sequencing or other advanced -omics [36]. Functional connectivity may also serve as a useful biomarker, especially if obtained through low-cost and more feasible technology such as EEG [37]. Unfortunately, the logistics and costs related to advanced imaging (e.g., MRI) might be prohibitive for widespread application during this phase. However, longitudinal specimen collection, especially saliva or urine, and the placement of stimulating electrodes to guide in-home therapy seems reasonable. A recent comprehensive review from the American Society of Neurorehabilitation highlights the challenges of developing effective biomarkers to guide rehabilitation [38]. The major value of biomarkers in this phase of stroke is to stratify patients to appropriate rehabilitation programs, tailor rehabilitation programs that prove initially ineffectual, and for counseling the patient, family, and environment on expected outcomes.

## Biomarkers Defined by Type of Stroke

Stroke is a heterogeneous disease in pathoetiology origins, as well as variables affecting recovery. Thus, this requires biomarker trials designed specifically for the type of stroke. Ongoing biomarker trials mainly focus on ischemic stroke. However, within this population are subpopulations of ischemic stroke patients who qualify for the recombinant tissue plasminogen activator and/or mechanical thrombectomy. As a result, treatment eligibility must also be considered. One current biomarker trial, BACTRAC, utilizes the thrombectomy procedure to collect blood at the time of stroke onset from the stroke-affected brain area [39]. This ongoing study is analyzing the blood distal to the clot for fluid biomarkers predictive of injury and/or recovery, with the within-patient proximal blood collection taken as a control standard. If specific biomarkers are identified for a subset of patients, and a rapid testing method developed, adjunctive therapies could be administered intra-arterially at the time of recanalization during mechanical thrombectomy. Thus, this creates a need for biomarkers for therapeutic development that dramatically differ from any other ischemic stroke patient subgroups, including all ischemic stroke patients who do not qualify for thrombectomy.

The main difference between ischemic and some hemorrhagic strokes is that in ischemia, the first 2 to 3 days after the onset of stroke are typically the worst for the patient, unless the patient has a large stroke where malignant edema occurs or there is early stroke re-occurrence. Thus, most important therapeutic decisions will be made in that time, which requires rapid biomarkers. In hemorrhagic conditions such as ICH and SAH, worsening conditions frequently occur in a delayed fashion. Figure 1 depicts the progression of hemorrhagic stroke injury as it differs from ischemic stroke. Both this and the differences in pathophysiology and clinical course between hemorrhagic and ischemic stroke thereby alter the intensive therapy and the post-stroke rehabilitation/recovery stages. Therefore, providing more time for long-term biomarkers in these latter conditions, as well as their predictive value for secondary injury, would be highly valuable to guide therapy and extend the window of intervention. Within hemorrhagic stroke populations, variations between ICH and SAH patients would suggest that, for example, ICH biomarkers should focus on mechanisms and treatment of brain edema, whereas SAH biomarkers could predict delayed vasoconstriction resulting in delayed cerebral ischemia (DCI). Finally and unfortunately, most ongoing biomarker trials exclude children with stroke, perhaps as pediatric stroke patients encounter varied etiology and are often not predictable within the first 24 h [40]. However, future neonatal- and pediatric-specific biomarker trials could have a significant impact as patients experience high rates of comorbidities and various degrees of learning deficits throughout childhood [41].

## Current Biomarker Trials for Stroke

Clinical trials for the discovery of diagnostic biomarkers that will help differentiate between stroke and stroke mimics (e.g., brain tumors, metabolic disorders, migraines, transient ischemic attacks), and between ischemic and hemorrhagic strokes, are underway. Table 1 provides a sampling of biomarker trials taking place throughout the world, predominantly in adults with ischemic stroke. While there are several different types of potential biomarkers, components found in blood are most commonly evaluated. However, due to the heterogeneity of comorbidities and types of stroke, it has proven difficult to identify biomarkers with adequate sensitivity and specificity, as previously mentioned [56]. Therefore, biomarker panels with a combination of markers related to inflammation, oxidative stress, blood–brain barrier disruption, and cell death may be of greater value, though they have yet to demonstrate sufficient accuracy in clinical use [52, 57–59].

**Table 1:** Brief overview of example biomarker clinical trials for stroke (ongoing and completed)

Clinical Trials (identifier)	Stroke type	Participant age	Sample type	Phase	Methods
Ischemia Care Biomarkers of Acute Stroke Etiology (BASE) (NCT02014896) [14] Identification of blood components that may differentiate between diverse stroke etiologies and clinical outcomes.	Ischemic	18+	Blood	Acute	Sample collection at 24 ± 6 and 48 h post-presentation. RNA expression measured by a gene array.
Biomarker Signature of Stroke Aetiology Study: The BIOSIGNAL Study (NCT02274727) Evaluate the predictive value of biomarkers to identify stroke etiology and risk of recurrence.	Ischemic	18+	Blood	Recurrent	Blood evaluation, cardiac rhythm monitors, ultrasound and MRI/MRA on admission and 1-year follow-up.
A novel biomarker-based prognostic score in acute ischemic stroke: The CoRisk score To validate a copeptin-based parsimonious score to predict unfavorable outcome 3 months after an acute ischemic stroke [42].	Ischemic	18+	Blood	Hyperacute and Recovery	Plasma drawn within 24 h of AIS; biomarker-based CoRisk score was well-calibrated in validation cohort, for 3-month prediction of disability and death.
Combined Approach to Lysis Utilizing Eptifibatide and recombinant tissue plasminogen activator in Acute Ischemic Stroke (CLEAR) (NCT00250991) [43] Examination of early genomic changes in whole blood prior to and following administration of recombinant tissue plasminogen activator.	Ischemic	18+	Blood	Hyperacute	Gene microarrays of white blood cells and whole blood at < 3, 5, and 24 h after stroke.
SMARTCapStroke Study: A Field Deployable Blood Test for Stroke (NCT02308605) Use of a biosensor array for the detection of purines produced following stroke.	Ischemic	All	Blood	Hyperacute and acute	Blood samples at admission and 24 h post-stroke are analyzed by an array of biosensors and electrodes that can be placed inside a vacutainer tube.
Blood And Clot Thrombectomy Registry And Collaboration (BACTRAC) (NCT03153683) [39] Collection of arterial blood and thrombus during a standard thrombectomy procedure for identifying novel biomarkers.	Ischemic	18+	Blood and Thrombus	Hyperacute	Blood proximal and distal to the thrombus, as well as the thrombus, are collected for gene expression and proteomics analysis.
Effect of Prior Atorvastatin Treatment on the Frequency of Hospital acquired Pneumonia and Evolution of Biomarkers in Patients with Acute Ischemic Stroke (Chinese Clinical Trial Regisrtry-ROC-17010633) [44]	Ischemic	18+	Blood	Acute	Blood samples collected upon ischemic infarct diagnosis by MRI.
Vitamin D and falls in people with stroke (Australian New Zealand Clinical Trials Registry CTRN12618000358246) Trajectory of Vitamin D and other biomarkers in the first 12 months after stroke and the relationship of these biomarkers with falls.	Ischemic	18+	Blood	Recovery	Evaluation of vitamin D and other biomarkers over 12 months related to falls, injury, and functional outcomes, including strength, balance, and walking.
Genetics of Ischemic Stroke Functional Outcome Network: GISCOME Identification of genetic loci associated with stroke risk and functional outcome.	Ischemic	18+	Blood	Recovery	Genome-wide association study (GWAS). Modified Rankin score (mRS) at 60–190 days was the most readily available outcome measure.
Predictors for Post-stroke Outcomes: Tel Aviv Brain Acute Stroke Cohort (TABASCO) (NCT01926691) [45] Identification of factors involved in post-stroke cognitive and functional decline.	Ischemic	50+	Blood and saliva	Recovery	Evaluation of genetic, inflammatory, and psychological markers acquired during acute phase to predict long-term cognition and function.
A prospective study of serum metabolites and risk of ischemic stroke Blood-based biomarkers and novel etiologic pathways of disease risk, by untargeted serum metabolomics profiling in ischemic stroke [46].	Ischemic	45–64	Blood	Pre-stroke	Two serum long-chain dicarboxylic acids, metabolic products of ω-oxidation of fatty acids, were linked with IS and cardioembolic stroke independent of known risk factors
Immunophenotyping of cerebrospinal fluid cells in ischaemic stroke Immune-cell profiling in CSF and peripheral blood of patients with acute ischemic stroke and healthy controls [47].	Ischemic and healthy	18+	Blood and CSF	Acute and recovery	Stroke induced slight increase in CSF immune cells without changes in immune cell subsets. Therefore, brain inflammation does not sufficiently reveal in CSF. CSF cell analysis not suitable biomarkers for possible immune therapies in stroke.
The Stroke-Chip Study [48] Develop a panel of biomarkers to differentiate between stroke and stroke mimics and between ischemic and hemorrhagic strokes.	Ischemic and hemorrhagic	18+	Blood	Hyperacute	Sample collection < 6 h post-presentation. Blood evaluation by antibody-based array.
The VITAL Study and Overall Pooled Analysis with VIPS Non-invasive Detection Device (NCT03148340) [49] Use of the VIPS device for the detection of severe stroke to improve the triage of patients.	Ischemic and hemorrhagic	18+	VIPS monitoring	Pre-stroke and hyperacute	VIPS device (emits low electromagnetic waves to detect fluid/brain damage) was used to differentiate between severe and minor strokes.
Helsinki Ultra-acute Stroke Biomarker Study (NCT02145663) [50] Development of biomarkers obtained in a prehospital setting to support early initiation of treatment.	Ischemic and hemorrhagic	18+	Blood	Pre-stroke	Blood samples collected by EMS personnel to identify ischemic and hemorrhagic strokes, TIAs, and stroke mimics.
Early Biomarkers of Stroke [51] Protein array was developed to identify biomarkers of acute stroke which can be used in conjunction with CT imaging for the identification of stroke subtype.	Ischemic and hemorrhagic	18+	Blood	Hyperacute	ELISA panel was developed as an adjunct to CT imaging for the differentiation between ischemic and hemorrhagic strokes within the first 6 h of symptom onset.
BRAIN (Biomarker Rapid Assessment of Ischemic iNjury) (NCT00206908) [52] Evaluation of a blood panel for the diagnosis or stroke vs. stroke mimics.	Ischemic and hemorrhagic	18+	Blood	Hyperacute and acute	Blood samples collected at symptom onset and up to 72 h (ischemic) or days 3 to 14 (hemorrhage).
Transcriptomic Signature of Vasospasm Consecutive to Sub-arachnoid Aneurismal Hemorrhage: VASOGENE (NCT01779713) [53] Development of biomarker to identify patients with aSAH who will develop arterial vasospasm.	Hemorrhagic	18+	Blood	Acute	Sample collection daily from admission to day 12. Blood evaluation of gene and miRNA expression.
The effect of melatonin on patients with hemorrhagic stroke (Iranian Registry of Clinical Trials 2016100530164N1) [54]	Hemorrhagic	All	Blood	Acute	S100B and CRP as a biomarker of neuronal injury will be assessed once on days 1 and 5 post ICU admission
EudraCT Number: 2007-002337-36 An open-labeled study of the cerebrospinal fluid pharmacokinetics of intravenous Kineret® in patients with subarachnoid hemorrhage.	SAH	All	CSF	Acute	Study of CSF pharmacokinetics of intravenous Kineret® in patients with an external ventricular drain inserted for clinical management
Baseline and On-statin Treatment Lipoprotein(a) Levels for Prediction of Cardiovascular Events [55] Meta-analysis of 7 clinical trials for the association of lipoprotein(a) levels and risk of cardiovascular events.	Not Specified	18+	Blood	Pre-stroke and recurrent	Measurement of lipoprotein(a) levels as a predictor of cardiovascular events or stroke.

An important aspect to take into consideration is the disease phase in which the biomarker is evaluated. As shown in Table 1, several trials collect the specimens in the hyperacute/acute phase of stroke. Many of these trials, such as the Biomarkers of Acute Stroke Etiology (BASE) trial, are attempting to identify biomarkers that will help diagnose stroke at the time of admission. However, it is important to note that while samples are collected during the hyperacute/acute phase, they are often subsequently frozen for later analysis. An improvement in the speed of laboratory technology would optimize biomarkers to be used in the clinical setting at the time of patient presentation. Other studies, while few, are evaluating biomarkers during the post-stroke recovery/rehabilitation phase, with some focusing on identifying biomarkers related to recurrent stroke. This is particularly relevant as the risk factor for recurrent stroke remains high, especially in patients with comorbidities [60]. Interestingly, the Tel Aviv Brain Acute Stroke Cohort (TABASCO) study examined a combination of genetic, inflammatory, imaging, and psychological markers during the acute phase to predict long-term cognitive and functional decline, with collection of both blood and saliva [61]. While TABASCO identified imaging abnormalities, motor function (i.e., balance, gait), and comorbidities as biomarkers for cognitive decline [45, 62, 63], blood and saliva results have yet to be published. Unfortunately, although a number of clinical trials continue, no blood biomarker or panel of biomarkers has significantly added to the diagnostics already obtained through physical examination and imaging [64].

## Designing Ideal Scenarios for Biomarker Development

The selection and development of biomarkers should be based on a clear understanding of the etiology of the given stroke subtypes, relevant complications, and the recovery phase. With that in mind, taking aneurysm SAH (aSAH) as an example, pathological injuries develop over weeks. Symptoms in which a practitioner could intervene would include initial severity of injury, secondary cerebral vasospasm (CV), and DCI. aSAH patients are routinely kept in the hospital for > 14 days to monitor for development of secondary complications. Clinically, it is difficult to predict whether a patient has CV and symptomatic ischemia because these patients are critically ill and the neurologic decline associated with delayed CV/DCI/infarction is not discernable from the initial critical state. Due to long-term functional impairments caused by CV/DCI/infarction, identifying diagnostic biomarkers to correlate with the severity of injury and prognostic biomarkers for the early prediction of CV, as well as diagnostic biomarkers for monitoring the rise, peak, and resolution of CV, could serve as clinically useful tools in the critical care management of aSAH patients. Furthermore, understanding the dynamic and temporal biomarker levels relative to the patient’s clinical course would shed important insight on aSAH pathophysiology and reveal novel putative therapeutic targets.

In terms of biospecimens, it is necessary to consider blood, CSF if available, buffy coat, saliva, and urine. It would be ideal to collect temporal samples at admission and at given period intervals (e.g., 6 h) for up to 14 days post-bleed, biospecimens collected as early as possible, including during transportation in the ambulance, dependent on the expertise of the first responders and ease of access. These temporal profiles could then be correlated to initial clinical and radiographic aSAH severity; the incidence, dynamics, and severity of CV; the incidence of DCI and/or infarction; mortality; and functional outcome scales at the time of discharge, through 12 months post-bleed. Comparing the rates of change would delineate the contribution of central versus peripheral clearance mechanisms fundamental to developing effective therapeutics. More specifically, changes in red blood cells and hemoglobin (Hb) levels tend to mirror the evolution of CV, although the mechanisms by which free Hb causes delayed arterial narrowing are multifactorial and poorly understood [65–67]. Possible mechanisms include neuronal apoptosis, increased endothelin-1, direct oxidative stress on smooth muscle cells, lipid peroxidation, and differential upregulation of related genes [67]. Further, the process by which blood metabolites are normally cleared from the subarachnoid space is unclear and may contribute to the inability to predict CV after aSAH. One advantageous aspect of aSAH is that many patients will have a shunt to drain CSF. This allows sample pairing (i.e., serum/CSF) collected at exact times from the same patient, to monitor the dynamics of a given biomarker, for example, when an early CSF biomarker appears in the blood. Appropriate control samples are also extremely valuable (and interestingly, sometimes more difficult for a center to obtain, especially with regard to CSF). For example, in the context of aSAH, the control biospecimens would consist of a single time point of simultaneously collected serum and CSF samples from acoustic neuroma, colloid cyst, or communicating hydrocephalus patients (non-aSAH). In all cases, the samples should be collected using standardized protocols and, if possible, by the same operating team.

Biostatisticians are vital for the investigation and should be included from the beginning as they provide the proper design analysis to identify biologically meaningful correlations. For longitudinal (repeated measures) models, general (continuous outcomes), and generalized (categorical/count outcomes), linear mixed models should be employed. These models should account for the intra-individual correlations in outcomes within subjects, missing data, and individuals having different values of measurements. Interaction terms between time and a given predictor (i.e., time × predictor) should be modeled to assess whether changes in the outcome (slope) differ across different levels of the biomarker. Finally, time-to-event measures (e.g., mortality) should be evaluated using Kaplan–Meier curves, log-rank tests for univariate analysis, and Cox regression for multivariable analysis. With the amount of data gathered, machine-learning technology could foster the development of prognostic and diagnostic biomarkers and provide a solid mechanistic understanding of the dynamics relative to clinical course. Along that line, the U.S. Food and Drug Administration (FDA) announced an effort to develop a regulatory framework for medical artificial intelligence (AI) algorithms that reflects the fact that these tools are continuously learning and evolving from experience gained in real-world clinical use [68, 69]. This biomarker-powered, self-learning engine may ultimately transform healthcare.

The development (and independent validation) of prognostic and diagnostic biomarkers also provides solid mechanistic understandings of pathology. All biomarker platforms should include methods to identify therapeutically targetable pathways. Furthermore, through various surgical procedures, acquisition of additional biospecimens could include blood at the site of infarction, blood clot, brain tissue, etc. In fact, more public/patient education and awareness would increase the number of brain and tissue donors, and optimized protocols should be in place to rapidly obtain post-mortem tissues. Combined efforts could support such infrastructure and allow investigators access to these biobanks. We suggest that the NIH StrokeNet, as well as international consortiums/associations (e.g., Hemoglobin After Intracranial Hemorrhage (HATCH) consortium [70]), include biospecimen banking while conducting clinical trials, although we acknowledge that funding would be required to support the infrastructure. Finally, the American Heart Association’s Precision Medicine Platform [71] would be an excellent shared database for the deposition of acquired data from shared biobanking consortiums.

## Developing Biomarkers from Preclinical Studies: from Bedside to Bench Then Back to Bedside

One intriguing opportunity that brings together clinicians and basic/translational stroke researchers is the idea of reverse translation by which novel clinical stroke biomarkers could be studied in animal models of stroke. In this way, clinical researchers cast a wide net to screen samples for factors that are impacted by stroke in an unbiased, hypothesis-free fashion, to be followed up by bench experimentation. For example, if a particular protein, cytokine, etc., is acutely elevated in many/most patients undergoing ischemic stroke, experiments could determine whether this also occurs in lab animals after experimental stroke, followed by gain- and loss-of-function, mechanistic, and therapeutic studies. The results of such preclinical investigations could be translated back to the clinic in the form of biomarker validation, novel drug or intervention development, and clinical trials. Indeed, during the translation of many promising preclinical stroke biomarkers and therapies has been fraught with failures [72], it is enticing to speculate that reverse translation approaches, grounded in clinical realities, may improve the chances of success.

Analogous to “drugability” in pharmacology, animal studies may also be used to conceptualize “biomarkerability.” The experimental scenario may allow for biomarker screening in well-defined experimental conditions [36], which can narrow the selection of biomarkers to be detected by ultrasensitive methods. For example, there are thousands of various microRNAs (miRNAs) across the body that control mRNA for brain-specific transcription factors, which may be present, albeit only a few copies, in body fluids following stroke. Genome-wide methods, such as next-generation sequencing, may exhibit insufficient sensitivity to detect biomarker-ready miRNAs. However, if miRNAs are identified based on analysis of infarcted tissue and verified in clinical samples, then sensitive methods such as droplet digital PCR could be developed to catch injury or repair-relevant miRNAs in body fluids—even single copies of particular miRNAs—which may become valuable biomarkers detectable rapidly even during the stroke phase.

## Concluding Remarks

In conclusion, there is momentum moving the field into a new frontier of stroke research—precision medicine. The effective use of biomarkers allows for more precise phenotyping of patients, monitoring of disease progression and response to therapy, and drug discovery. This endeavor will require international coordination of research efforts, data, and resources. There are several limitations to consider that will require unique approaches, such as data sharing across different governing ethics boards and countries, data analysis, consistency of shared data elements and documentation, cost of sample collection and analysis, and reverse and forward translation of research findings. An approach to maneuver around these challenges will require coordination and collaboration, as well as research efforts from basic scientists, clinical researchers, clinicians, policy-makers, community, and industry to use funds efficiently and reduce redundancy. Nonetheless, the power to better shape the recovery of millions of stroke survivors each year demands that we as a field harness new technologies to design optimal biomarker trials that support the development of new neurotherapeutic interventions. The new FDA medical AI regulatory effort, coupled with the AHA Precision Medicine Platform, may also transform healthcare into a biomarker-powered, self-learning engine capable of providing optimal service to patients, including those with stroke, on a globally accessible level.
